# A Transfer Learning Approach to Correct the Temporal Performance Drift of Clinical Prediction Models: Retrospective Cohort Study

**DOI:** 10.2196/38053

**Published:** 2022-11-09

**Authors:** Xiangzhou Zhang, Yunfei Xue, Xinyu Su, Shaoyong Chen, Kang Liu, Weiqi Chen, Mei Liu, Yong Hu

**Affiliations:** 1 Big Data Decision Institute Jinan University Guangzhou China; 2 College of Information Science and Technology Jinan University Guangzhou China; 3 School of Management Jinan University Guangzhou China; 4 Division of Medical Informatics Department of Internal Medicine University of Kansas Medical Center Kansas City, KS United States

**Keywords:** transfer learning, clinical prediction model, performance drift, concept drift, acute kidney injury

## Abstract

**Background:**

Clinical prediction models suffer from performance drift as the patient population shifts over time. There is a great need for model updating approaches or modeling frameworks that can effectively use the old and new data.

**Objective:**

Based on the paradigm of transfer learning, we aimed to develop a novel modeling framework that transfers old knowledge to the new environment for prediction tasks, and contributes to performance drift correction.

**Methods:**

The proposed predictive modeling framework maintains a logistic regression–based stacking ensemble of 2 gradient boosting machine (GBM) models representing old and new knowledge learned from old and new data, respectively (referred to as transfer learning gradient boosting machine [TransferGBM]). The ensemble learning procedure can dynamically balance the old and new knowledge. Using 2010-2017 electronic health record data on a retrospective cohort of 141,696 patients, we validated TransferGBM for hospital-acquired acute kidney injury prediction.

**Results:**

The baseline models (ie, transported models) that were trained on 2010 and 2011 data showed significant performance drift in the temporal validation with 2012-2017 data. Refitting these models using updated samples resulted in performance gains in nearly all cases. The proposed TransferGBM model succeeded in achieving uniformly better performance than the refitted models.

**Conclusions:**

Under the scenario of population shift, incorporating new knowledge while preserving old knowledge is essential for maintaining stable performance. Transfer learning combined with stacking ensemble learning can help achieve a balance of old and new knowledge in a flexible and adaptive way, even in the case of insufficient new data.

## Introduction

Clinical risk prediction models can provide decision-making support on therapeutic interventions and resource allocation, and thus can improve patient outcomes and reduce medical costs [1]. Along with the increasing availability and volume of electronic health record (EHR) data, these models are evolving from rule-based to data-driven probability-based tools, for example, machine learning–based patient outcome prediction models [2]. One of the critical challenges is performance drift over time, which results from either gradual or quick data shifts in the patient population, such as changing patient outcome rate, evolving clinical practices, and improving measurement accuracy [3].

To correct temporal performance drift, a range of model updating approaches are available, including recalibration, model-specific adaptation (eg, reweighting the leaf nodes of each tree in a random forest [RF] model and an incremental learning method for a neural network model), model extension (eg, incorporating new predictors), and full model refitting [1]. These updating approaches vary in analytical complexity, old data and updated sample requirements, and computational demands. Usually, full model refitting is not the leading choice, especially in clinical use, owing to the risk of overfitting when new (and often smaller) data are used alone, while old data are completely discarded [1]. The essence of model updating is to create models that are constantly updated and adapted to the new incoming data, while balancing between both new and old knowledge [4-7].

Acute kidney injury (AKI) is a potentially life-threatening clinical syndrome, for which the only effective treatments are supportive care and dialysis, and it affects 10%-15% of all inpatients and more than 50% of critical care patients, and results in high mortality [8,9]. For AKI prediction, Davis et al [2] developed 7 common regression and machine learning models, and found that discrimination performance declines were statistically significant but small for all models. Since they collected data solely from US Department of Veterans Affairs hospitals, it is not a typical scenario of population drift. Using data collected from Royal London Hospital, which hosts Europe’s largest kidney treatment facility, Haines et al [10] developed risk prediction models for AKI after trauma, with the area under the receiver operating characteristic curve (AUROC) declining from 0.77 (0.72-0.81) in the development set (February 2012 to October 2014) to 0.70 (0.64-0.77) in the validation set (November 2014 to May 2016), and significant temporal performance drift.

In this study, we developed a clinical risk prediction model for hospital-acquired AKI. The model has been named transfer learning gradient boosting machine (TransferGBM), which is based on a transfer learning paradigm and maintains a stacking ensemble of 2 base gradient boosting machine (GBM) learners. Transfer learning has been proven to be one of the most effective ways to deal with data scarcity (eg, in the scenario where new data are not sufficient or available at a low cost) and data distribution discrepancies in many areas [11-17]. Transfer learning aims to selectively reuse data or knowledge from the source domain to assist the modeling process on the target domain, and it can be used to tackle the performance drift problem by regarding the old data as the source domain and the new data as the target domain. Since existing transfer learning approaches focus on optimizing performance only in the target domain, we still need a well-designed mechanism to incorporate and balance the old and new knowledge learned from the source and target domains.

## Methods

### Definition of AKI

According to the Kidney Disease Improving Global Outcomes (KDIGO) clinical practice guidelines for AKI, we adopted serum creatinine (SCr)-based criteria to stage the severity of AKI [18]. We did not use urine output to define AKI because it is less likely to be accurate outside the critical care environment [19,20]. Mild AKI (“AKI stage 1”) is defined as an increase in SCr of 1.5 to 1.9 times the baseline value within 7 days or an increase in SCr to 0.3 mg/dL (26.5 μmol/L) or more within 48 hours. The baseline creatinine value is defined as the most recent SCr if available; otherwise, it is the admission SCr. Moderate AKI (“AKI stage 2”) is defined as an increase in SCr of 2.0 to 2.9 times the baseline value within 7 days. The most severe AKI (“AKI stage 3”) is defined as an increase in SCr of 3.0 or more times the baseline value within 7 days or an increase in SCr to 4 mg/dL (353.5 μmol/L) after an acute increase of at least 0.3 mg/dL within 48 h or initiation of renal replacement therapy.

### Study Cohort

The study constructed a retrospective cohort using deidentified EHR data from 2010 to 2017 in the University of Kansas Medical Center. The data have been used in a previous study [20] including a total of 141,696 adult patients (121,537 non-AKI patients; 20,159 any AKI patients; 3150 AKI stage ≥2 patients; and 1491 AKI stage 3 patients). To reflect the inpatient population shift, patients enrolled in different years were regarded as distinct individuals (ie, we handled the data at the patient-encounter level).

As shown in Table 1, the proportion of elderly patients (ie, age ≥65) generally increased every year, from 31.7% in 2010 to 36.5% in 2017. The proportion of patients between the ages of 46 and 55 years decreased every year, while the proportion of patients in other age groups remained the same. The ratio of male to female patients did not change much over time, and was basically maintained at 1:1. The proportion of White patients always ranked first, accounting for more than 70% of the total number of samples in each year, while the proportion of Native Hawaiians was the least (only 0.1%). Only the proportion of patients from different ethnicities remained stable over time, without obvious changes. The proportion of African Americans was more in 2010 than in all other years, and the proportion of White patients was slightly less in 2010 than in all other years. In addition, the incidence of AKI (any AKI) showed a clear downward trend, from 16.9% in 2010 to 12.8% in 2017.

**Table 1:** Demographic information.

Feature		Year							
		2010 (N=14,946)	2011 (N=15,422)	2012 (N=16,682)	2013 (N=17,450)	2014 (N=18,701)	2015 (N=20,094)	2016 (N=20,399)	2017 (N=18,002)
**Age group (years), n (%)**									
	18-25	869 (5.8)	886 (5.7)	923 (5.5)	918 (5.3)	1077 (5.8)	1082 (5.4)	1086 (5.3)	1001 (5.6)
	26-35	1290 (8.6)	1275 (8.3)	1468 (8.7)	1567 (9.0)	1717 (9.7)	1814 (9.0)	1823 (8.9)	1664 (9.2)
	36-45	1640 (11.0)	1727 (11.2)	1696 (10.2)	1861 (10.7)	1819 (9.7)	2136 (10.6)	2196 (10.8)	1919 (10.7)
	46-55	3025 (20.2)	2998 (19.4)	3203 (19.2)	3133 (19.0)	3150 (16.8)	3482 (17.3)	3259 (16.0)	2762 (15.3)
	56-65	3383 (22.6)	3659 (23.7)	3951 (23.7)	4161 (23.8)	4558 (24.4)	4897 (24.4)	4840 (23.7)	4088 (22.7)
	>65	4739 (31.7)	4877 (31.6)	5441 (32.6)	5810 (33.3)	6380 (34.1)	6683 (33.3)	7195 (35.3)	6568 (36.5)
**Sex, n (%)**									
	Male	7547 (50.5)	7635 (49.5)	8432 (50.5)	8640 (49.5)	9307 (49.8)	10,114 (50.3)	10,250 (50.2)	9045 (50.2)
	Female	7399 (49.5)	7787 (50.5)	8250 (49.5)	8810 (50.5)	9394 (50.2)	9980 (49.7)	10,149 (49.8)	8957 (49.8)
**Race, n (%)**									
	American Indian	53 (0.4)	52 (0.3)	46 (0.3)	79 (0.5)	68 (0.4)	87 (0.4)	80 (0.4)	63 (0.3)
	Asian	125 (0.8)	128 (0.8)	153 (0.9)	167 (1.0)	210 (1.1)	184 (0.9)	254 (1.2)	149 (0.8)
	African American	2286 (15.3)	2240 (14.5)	2255 (13.5)	2510 (13.4)	2685 (14.4)	2883 (14.3)	2896 (14.2)	2614 (14.5)
	Native Hawaiian	11 (0.1)	20 (0.1)	9 (0.1)	9 (0.1)	15 (0.1)	10 (0.1)	18 (0.1)	14 (0.1)
	White	10,915 (72.9)	11,485 (74.5)	12,691 (76.1)	13,331 (76.4)	14,322 (76.6)	15,378 (76.5)	15,522 (76.1)	13,689 (76.0)
	Multiple races	22 (0.1)	24 (0.2)	51 (0.3)	46 (0.3)	53 (0.3)	38 (0.2)	41 (0.2)	28 (0.2)
	Others	1534 (10.3)	1473 (9.6)	1477 (8.9)	1308 (7.5)	1348 (7.2)	1514 (7.5)	1588 (7.8)	1445 (8.0)
**Label, n (%)**									
	Non-AKI^a^	12,414 (83.1)	12,937 (83.9)	14,097 (84.5)	15,124 (86.7)	16,165 (86.4)	17,435 (86.8)	17,660 (86.6)	15,705 (87.2)
	Any AKI	2532 (16.9)	2485 (16.1)	2585 (15.5)	2326 (13.3)	2536 (13.6)	2659 (13.2)	2739 (13.4)	2297 (12.8)
	AKI stage ≥2	353 (2.4)	356 (2.3)	359 (2.1)	371 (2.1)	419 (2.2)	471 (2.3)	444 (2.2)	377 (2.1)
	AKI stage 3	146 (1.0)	149 (1.0)	171 (1.0)	184 (1.1)	187 (1.0)	241 (1.2)	219 (1.1)	194 (1.1)

^a^AKI: acute kidney injury.

### Data Preprocessing

For each patient, we collected all currently populated variables in the PCORNet common data model (CDM) schema, including demographic details (ie, age, gender, and race); structured clinical variables, including comorbidities (International Classification of Diseases-9 and International Classification of Diseases-10 codes), procedures (International Classification of Diseases and Current Procedural Terminology codes), laboratory tests (Logical Observation Identifiers Names and Codes), and medications (RxNorm and National Drug Code); and several vital signs (eg, blood pressure, height, weight, and BMI) [21]. All variables are time stamped, and each sample in the data set is represented by a series of clinical observation vectors aggregated on a daily basis. Therefore, the feature set formed by the data before or on day *t* can be used to predict AKI within days [*t*, *t*+1] for 24-h prediction (or within days [*t*+1, *t*+2] for 48-h prediction).

We preprocessed the data set as follows. First, for numerical features, such as laboratory measurement values and vital signs, we systematically removed the extreme values exceeding 1% and 99%. Second, we performed one-hot coding on categorical variables, such as diagnosis and procedure, to convert them into binary representations. Third, for medication codes, we converted data to cumulative exposure days before the prediction time rather than binary representations. Fourth, the most recent measurement value was chosen when repeated records were available within a certain time interval. Fifth, we used the “sample-and-hold” method to retrieve earlier available measurement values, when measurements were missing for a certain time span. Sixth, we introduced additional features, such as daily blood pressure trend or length of hospital stay, which have been shown to be useful for predicting AKI [22]. Seventh, we excluded all forms of SCr and blood urea nitrogen as they have a high correlation with AKI diagnosis and are not suitable for continuous prediction. Finally, a total of 28,306 features were obtained for model development.

**Figure 1:**
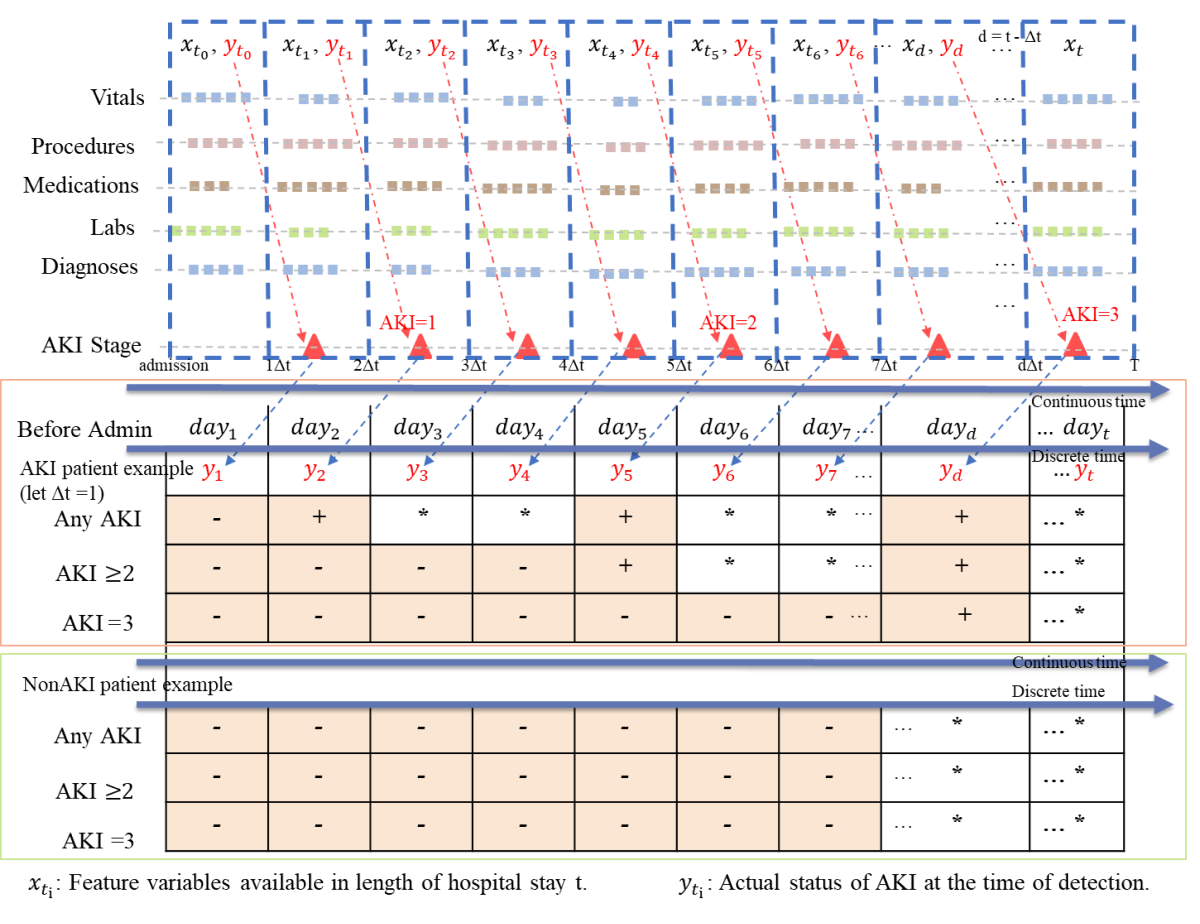
Data processing strategy based on the discrete-time survival framework. The red triangle represents the actual stage of acute kidney injury (AKI). "Δt" indicates the prediction time in advance, “−“ indicates negative sample, “+” indicates positive sample, and “*” indicates excluded sample.

We adopted the discrete-time survival framework [23] to preprocess the time-stamped EHR data, as shown in Figure 1. We divided the patient’s entire stay period into *L* nonoverlapping daily windows (ie, *L*=Δ*t*, 2Δ*t*, ..., *T*), where *T* is the length of hospital stay or a specific censor point. Based on expert knowledge, we chose a censor point *T*=7, which represents 7 days since admission. The interval value Δ*t* is the prediction window selected according to clinical needs. For example, Δ*t*=1 means 1-day (24-h) prediction and Δ*t*=2 means 2-day (48-h) prediction. We would use all available data up to time *t*-Δ*t* to predict AKI risk in time *t*. We treated the data corresponding to the AKI-onset day as positive samples based on the criteria of different prediction tasks, while the data after the first positive sample day and between different AKI-stage days were discarded since we could not judge the true AKI stages within these periods because physicians might have intervened and the patient’s condition might have improved. All remaining data were regarded as negative samples. For patients who never developed AKI during hospitalization, all available data within 7 days since admission were used to construct negative samples, and other data after 7 days since admission were discarded for the sake of alleviating data imbalance. Under the discrete-time survival framework, we can train a model more in line with real-world clinical practice, where the rolling prediction of AKI risk for a patient on a daily basis is essential [24].

### TransferGBM Modeling Framework

**Figure 2:**
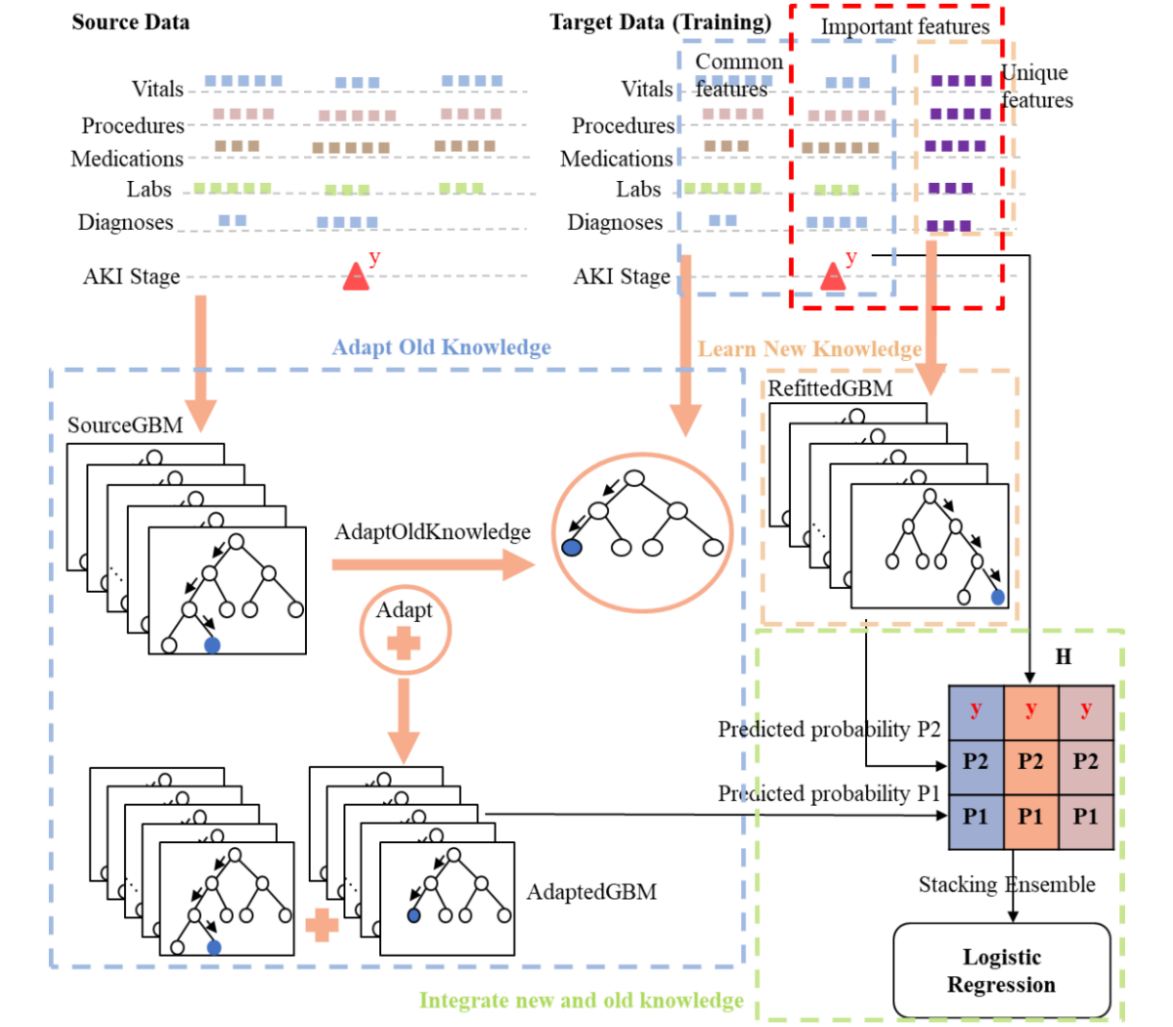
Illustration of the TransferGBM modeling framework. AdaptedGBM: adapted gradient boosting machine; AKI: acute kidney injury; RefittedGBM: refitted gradient boosting machine; SourceGBM: source gradient boosting machine; TransferGBM: transfer learning gradient boosting machine.

To correct temporal performance drift, we propose a transfer learning–based modeling framework named TransferGBM, as shown in Figure 2.

From the perspective of the transfer learning paradigm, we regard the old data as the source domain or source data, and the new data as the target domain or target data. We designed TransferGBM based on several fundamental ideas. First, the base learner is GBM, which has been applied in a wide range of clinical prediction modeling studies [25,26]. GBM has been chosen because (1) it is robust to high-dimensional and collinearity data, (2) it can automatically process missing values, and (3) it embeds a unique feature selection scheme in the model training process, making its output more interpretable [20,27]. Second, we treated the new and old data in different ways, with 2 independent GBM models representing the new and old knowledge, respectively. Third, we transferred old knowledge to the target domain while balancing new and old knowledge in the prediction through an ensemble of the above 2 GBM models. Fourth, we periodically updated the 2 GBM models and their relative weights in the prediction function using target data, in order to adapt to the changing data distribution.

The TransferGBM modeling framework included 5 steps. First, we constructed the source model (ie, source gradient boosting machine [SourceGBM]) using all source data, with a cross-validation–based procedure searching the optimal feature engineering scheme and hyperparameters of GBM (eg, depth of trees, learning rate, minimal child weight, and early stopping). Second, we applied the above optimal feature engineering scheme to the target data and then adapted SourceGBM to the processed target data using the built-in incremental learning mechanism and obtained the adapted model (ie, adapted gradient boosting machine [AdaptedGBM]). Third, we constructed the target model (ie, refitted gradient boosting machine [RefittedGBM]) using the original development set of the target domain while reusing the optimal feature engineering scheme and hyperparameters of GBM from SourceGBM. Fourth, we constructed the predicted probability value matrix for stacking ensemble learning [28], by combining the predicted probability values of AdaptedGBM and RefittedGBM for each sample from the target domain’s development set and the true label of the sample into a vector, and pooling all vectors into a matrix *H*. Fifth, we applied the stacking ensemble learning method with the logistic regression (LR) learner to the matrix *H* to obtain the final prediction model, which integrated the old and new knowledge from the AdaptedGBM and RefittedGBM models, respectively.

**Figure 3:**
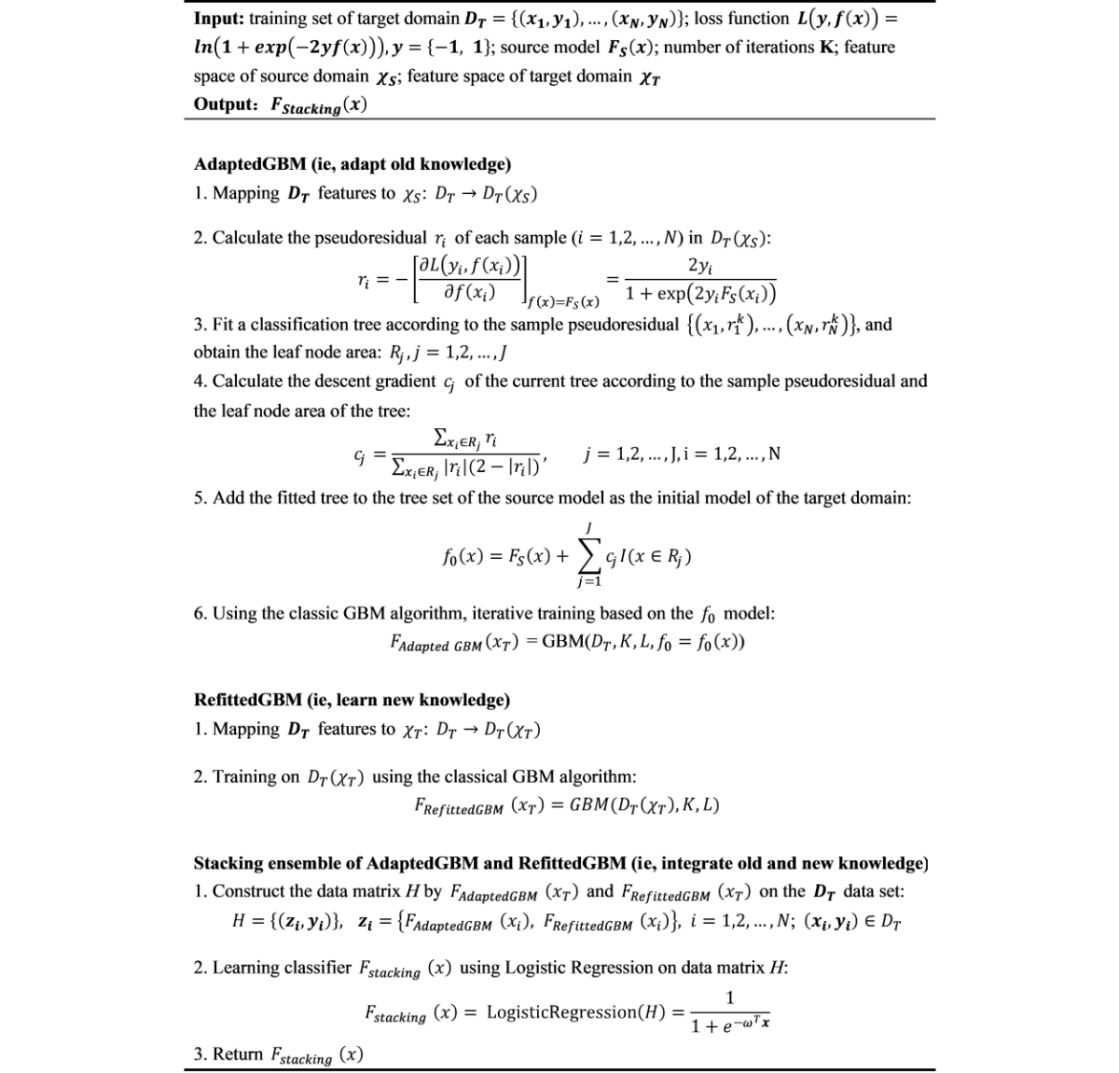
Pseudocode of the TransferGBM modeling framework. AdaptedGBM: adapted gradient boosting machine; GBM: gradient boosting machine; RefittedGBM: refitted gradient boosting machine; TransferGBM: transfer learning gradient boosting machine.

From the viewpoint of the target domain, the modeling procedure involved 3 distinct sets of features, including (1) the common features that indicate the intersection of the source and target domain features, (2) the unique features that indicate the features belonging to the target domain but not the source domain, and (3) the important features selected by the GBM learner from the target data. When we adapted SourceGBM, we used the common features extracted from the target data combined with missing values of source domain–specific features, so that we could transfer the old knowledge of SourceGBM to the target domain. Considering the value of the target domain–specific knowledge (ie, the new knowledge), we allowed the GBM learner to select the most important features from both the common and unique features of the target data, so that we could obtain the new knowledge of the target domain without constrains on the feature space. The pseudocode of the TransferGBM modeling framework is shown in Figure 3.

### Experimental Design

We designed the following 3 prediction tasks: any AKI prediction (ie, AKI stage ≥1), moderate-to-severe AKI prediction (ie, AKI stage ≥2), and severe AKI prediction (AKI stage 3). For any AKI prediction, the prediction window was set to 48 hours, while it was 24 hours for the other 2 tasks, according to general clinical needs.

We pooled the 2010 and 2011 data, and used them as old data (ie, a fixed source domain). The data from 2012 to 2017 were used as new data independently, yielding 6 target domains. We applied stratified random sampling to the source and target domain independently, with division into a development set (80%) and a validation set (20%). We tuned the hyperparameters of GBM, including depth of trees (2-10), learning rate (0.01-0.1), minimal child weight (1-10), and number of trees determined by early stopping, on the training set using 10-fold cross-validation. We measured model performance in terms of the AUROC [29], with a mean value from the 95% CI.

It should be noted that the performance of SourceGBM on the target domain’s validation set indicated temporal validation and the performance of RefittedGBM (trained using the target domain’s development set) on the target domain’s validation set indicated internal validation. To validate TransferGBM, we first explored whether there was performance drift over time and then whether TransferGBM could maintain performance.

### Ethical Considerations

The study did not require approval from an institutional review board because the data used met the de-identification criteria specified in the Health Insurance Portability and Accountability Act Privacy Rule [30]. The HERON Data Request Oversight Committee approved the data request.

## Results

### Base Model Selection

**Figure 4:**
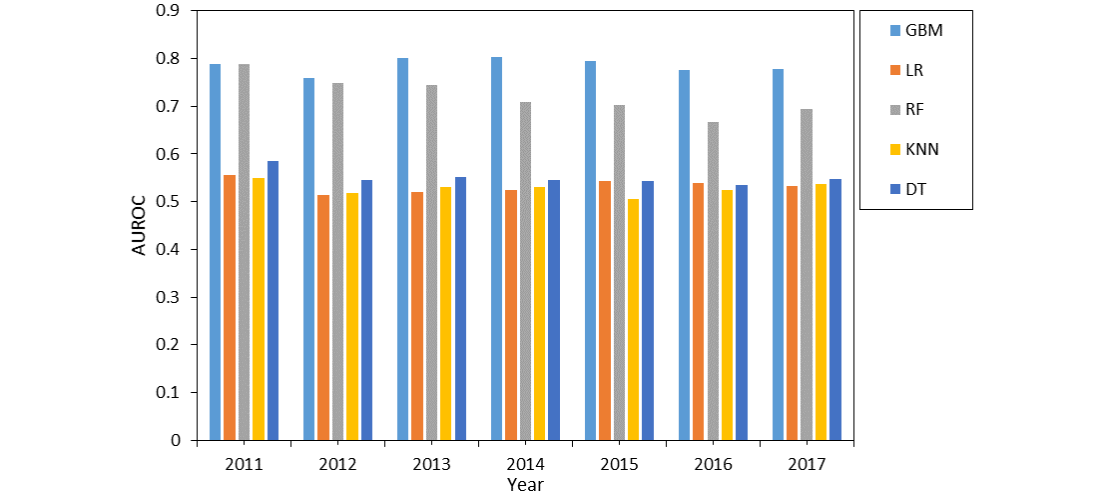
Internal validation of different machine learning models. AUROC: area under the receiver operating characteristic curve; DT: decision tree; GBM: gradient boosting machine; KNN: K-nearest neighbor; LR: logistic regression; RF: random forest.

We examined 5 common machine learning models based on 5-fold cross-validation on each year’s data for any AKI prediction. These models included LR, decision tree (DT), RF, K-nearest neighbor (KNN), and GBM. The model parameters were customized as shown in Table 2, in addition to the default parameters provided in the scikit-learn package [31]. The AUROC performances of the 5 models’ internal validations in different years are shown in Figure 4. The AUROCs of both GBM and RF reached 0.7 or above, indicating that these models had a certain predictive ability for AKI, while the performances of the other 3 models (DT, LR, and KNN) were generally poor. Given that GBM performed the best, we chose it as a base learner in the subsequent experiments.

**Table 2:** Model parameter setting.

Model	Parameter setting (except defaults)
Gradient boosting machine (XGBoost)	Tune the hyperparameters (depth of trees: 2-10; learning rate: 0.01-0.1; minimal child weight: 1-10) within the development set based on 10-fold cross-validation
Logistic regression	penalty=“L2;” max_iter=300; C=3.0
Random forest	n_estimators=400; bootstrap=True
K-nearest neighbor	n_neighbors=40
Decision tree	criterion=“entropy”

### Performance Shift Over Time

**Figure 5:**
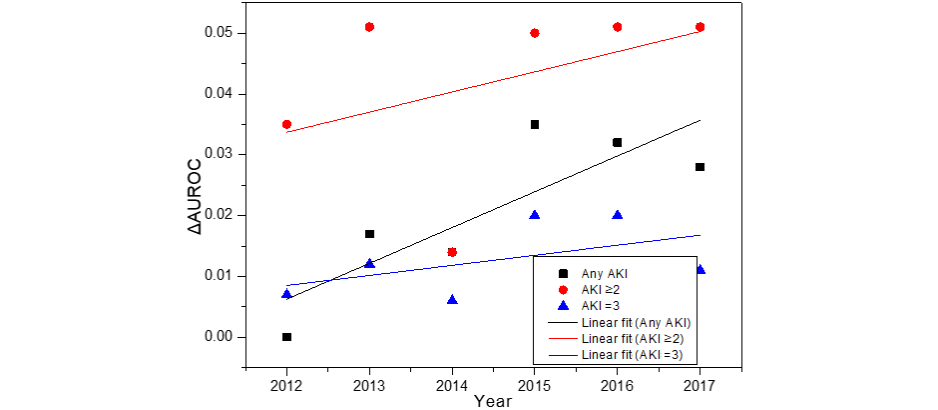
Performance gain by refitting the model. AKI: acute kidney injury; AUROC: area under the receiver operating characteristic curve.

Figure 5 depicts the AUROC gain (ie, ΔAUROC) between the internal validation of RefittedGBM relative to the temporal validation of SourceGBM across 3 prediction tasks. The ΔAUROC shows a linear growth trend over time, implying that the transported model (ie, direct transport of SourceGBM to the target domain without any adaptation) was not the best choice for new data due to the change in data distribution over time. From another point of view, the performance gain was within 0.051, implying that the transported model still contained some general knowledge that can be reused in the new data.

### Performance Validation of TransferGBM

TransferGBM maintained a stacking ensemble of 2 GBM models representing new and old knowledge learned from new and old data, respectively, with the former trained using data from 2010 and 2011, and the latter trained using the updated data of each year from 2012 to 2017. Using the validation set of the target domain from 2012 to 2017, we compared model performance between TransferGBM, transported gradient boosting machine (TransportedGBM, ie, direct transport of SourceGBM to the target domain without any adaptation), and RefittedGBM (ie, refitting SourceGBM using the target domain data). To better simulate the process of EHR accumulation in clinical applications, we further investigated different sizes of the available training set (ie, updated data) ranging from 25% to 100% of the target domain’s development set via stratified random sampling without replacement. Multimedia Appendix 1 shows the performance in terms of AUROC (95% CI) of TransportedGBM, RefittedGBM, and TransferGBM across different target years and different training set sizes for 3 prediction tasks.

**Figure 6:**
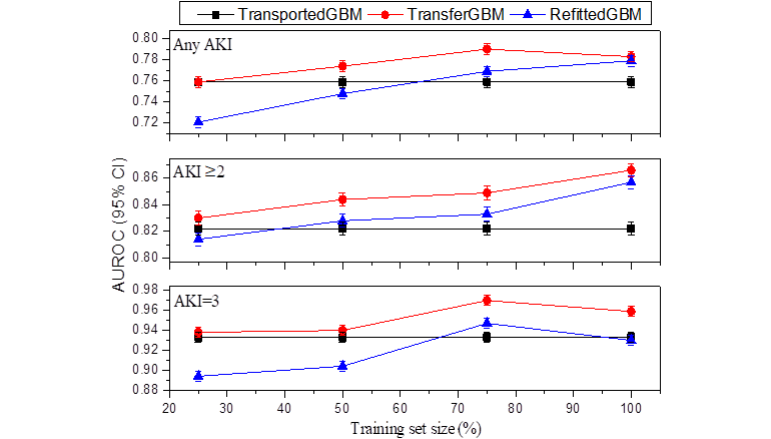
Impact of training set size on performance (target year 2012). AKI: acute kidney injury; AUROC: area under the receiver operating characteristic curve; RefittedGBM: refitted gradient boosting machine; TransferGBM: transfer learning gradient boosting machine; TransportedGBM: transported gradient boosting machine.

We assessed the impact of different sizes of available training sets on model performance from the perspective of modeling framework selection. Figure 6 illustrates the case of the target year 2012 as an example. The performance of TransportedGBM was better than that of RefittedGBM when the training set size was small. As the amount of training data increased, RefittedGBM gradually improved and finally outperformed TransportedGBM. Overall, regardless of the size of the available training set, the performance of TransferGBM was always better than that of TransportedGBM and RefittedGBM.

**Figure 7:**
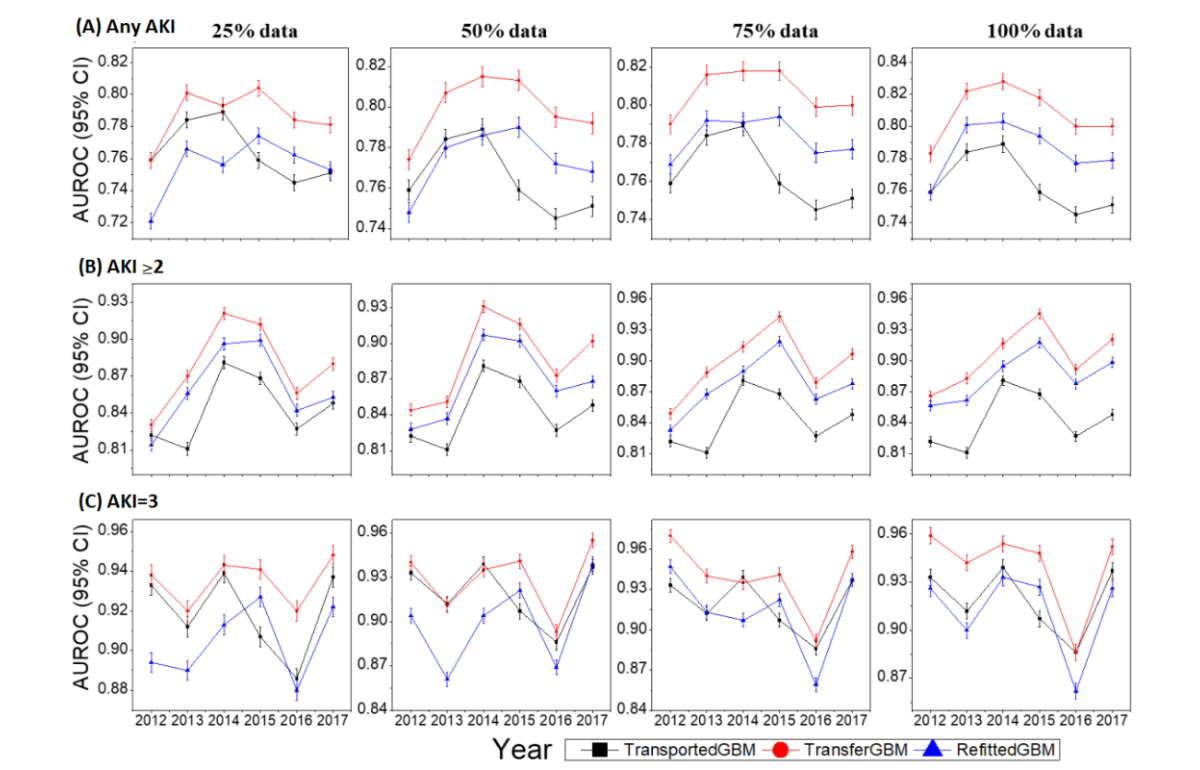
Joint impact of training set size and data distribution shift on performance. AKI: acute kidney injury; AUROC: area under the receiver operating characteristic curve; RefittedGBM: refitted gradient boosting machine; TransferGBM: transfer learning gradient boosting machine; TransportedGBM: transported gradient boosting machine.

Next, we investigated the joint impact of training set size and data distribution shift on model performance regarding the modeling framework selection, as shown in Figure 7.

For any AKI prediction, when the training set size was 25%, TransportedGBM outperformed RefittedGBM in the first 3 years (from 2012 to 2014). However, in the subsequent 3 years (from 2015 to 2017), the prediction of TransportedGBM rapidly declined, and it underperformed RefittedGBM. During the whole 6 years, TransferGBM consistently outperformed TransportedGBM and RefittedGBM, with the AUROC ranging from 0.759 (95% CI 0.732-0.766) to 0.804 (95% CI 0.778-0.812), and an average AUROC gain of 0.03 compared to RefittedGBM and 0.02 compared to TransportedGBM. When the training set size was 100%, RefittedGBM significantly outperformed TransportedGBM over all 6 years, but still underperformed TransferGBM. The AUROC of TransferGBM ranged from 0.783 (95% CI 0.757-0.792) to 0.828 (95% CI 0.802-0.834), with an average AUROC gain of 0.04 compared to RefittedGBM and 0.02 compared to TransportedGBM.

For AKI stage ≥2 prediction, even though the training set size was only 25%, RefittedGBM outperformed TransportedGBM (except for target year 2012), and a larger training set was associated with better prediction. This means that the data distribution of the target domain was significantly different from that of the source domain, and directly transporting an external model into the target domain was not a wise choice. Again, TransferGBM was the best model among the 3 models, regardless of the training set size and target year. The AUROC of TransferGBM ranged from 0.830 (95% CI 0.795-0.851) to 0.921 (95% CI 0.893-0.932) when the training set size was 25%, and ranged from 0.866 (95% CI 0.835-0.877) to 0.946 (95% CI 0.920-0.959) when the training set size was 100%.

For AKI stage 3 prediction, when the training set size was 25% or 50%, RefittedGBM significantly underperformed TransportedGBM in the first 3 years (from 2012 to 2014), but the prediction became close in the subsequent 3 years (from 2015 to 2017). When the training set size was 50% or 100%, RefittedGBM and TransportedGBM performed very close to each other. This result implies that direct transportation of an external model was a good choice (ie, there is no need to refit the model, especially when training data on the target domain is not sufficient). TransferGBM was still the best model, and the AUROC ranged from 0.920 (95% CI 0.890-0.936) to 0.948 (95% CI 0.921-0.962) when the training set size was 25%, and ranged from 0.866 (95% CI 0.854-0.911) to 0.959 (95% CI 0.932-0.973) when the training set size was 100%.

## Discussion

### Principal Findings

Experimental results showed that TransferGBM can consistently outperform TransportedGBM and RefittedGBM, regardless of the amount of available training data from the target domain. We also confirmed that old data are important, and should not be discarded, especially in the case of insufficient new data. There exist differences between old and new knowledge, and thus, there is a need to achieve balance.

With regard to the candidate base learners for the proposed transfer learning–based modeling framework, we considered several commonly used linear and nonlinear machine learning algorithms, and among them, RF has good robustness to overfitting and high-dimensional feature variables [32,33]. XGBoost can consider multiple potentially relevant predictors simultaneously and can handle potentially nonlinear correlations [34-36]. DT is a nonparametric learning algorithm with fast computation and accuracy, can handle continuous and type fields, and is very suitable for high-dimensional data [32]. LR is a linear algorithm that is very suitable for sparse data sets, and the model performance remains stable when only a few variables in the model are valuable predictors. KNN is simple to implement, does not require a data training process, and is very suitable for high-dimensional data. According to the experiment results, the XGBoost algorithm had superior performance. The performance of RF was very close to that of XGBoost, and both were tree-based ensemble approaches. DT may ignore the correlation between variables and experience some large noise, resulting in very poor model performance [33]. The poor performance of LR might be due to the nonlinear correlation between AKI risk factors. KNN may be affected by a large amount of noise in the EHR data, resulting in very poor performance.

The choice of TransportedGBM, RefittedGBM, or TransferGBM depends on or is affected by the actual situation regarding data distribution, modeling cost, available training data from the target domain, etc. TransportedGBM is trained on source data and then is directly applied to the target data without any adaptation and additional cost, which is appropriate for clinical scenarios where the distribution between the source and target domains is very similar. When the distribution is not similar, RefittedGBM would be a better choice than TransportedGBM, and it only requires refitting of the model on the target data, except for the requirement of sufficient training data from the target domain. TransferGBM is no doubt a more complicated solution, which needs to adapt an existing model, refit a new model, and construct an ensemble of these 2 models. This makes TransferGBM more suitable for clinical scenarios where the distribution of the source domain is partially similar to that of the target domain or where the degree of similarity changes significantly.

With regard to the adaptiveness of TransferGBM, it is clear that TransferGBM is a flexible and adaptive extension to the combination of AdaptedGBM and RefittedGBM (AdaptedGBM is obtained by updating TransportedGBM/SourceGBM to the target domain). This also means that TransferGBM might degrade to AdaptedGBM or RefittedGBM due to the stacking ensemble learning mechanism under certain situations. Taking some extreme cases as examples, when the target domain is under the same distribution as the source domain, TransferGBM would degrade to AdaptedGBM and even TransportedGBM since there is little change after updating the model with new data from the target domain. On the contrary, when the target domain is under a distribution completely different from the source domain, TransferGBM would degrade to RefittedGBM, since in this case, AdaptedGBM would be almost useless, and even negative and suppressed under the stacking ensemble learning process. In most cases that TransferGBM is designed for, that is, when the distributions of the source and target domains are more or less similar but not completely different, TransferGBM would adaptively achieve a balance between AdaptedGBM and RefittedGBM.

### Motivations

Conventionally, transfer learning is applied to the scenario of data scarcity and distribution disparity, with the underlying idea of selectively reusing data or knowledge from the source domain to assist the modeling process on the target domain. As for the scenario of temporal performance drift, we proposed to regard the old data as the source domain and the new data as the target domain, which might make transfer learning suitable, and we attempted to confirm its effectiveness.

We believe that transfer learning can provide insights from another perspective for correcting temporal performance drift, compared to common approaches such as recalibration and incremental training. For example, when the data distribution significantly changes, transfer learning can immediately discard the old knowledge/model and reselect a new suitable training sample from the source domain to learn, while incremental training suffers from slow progressive adaptation.

Since the primary objective of our study was not to build a high-performance AKI prediction model under the common modeling scenario, we divided the data into different years and adopted a simple and clear modeling process without comprehensive feature engineering, class balancing, hyperparameter searching, etc.

### Limitations

There are several limitations associated with our study. First, we used retrospective data in model training and validations, and had not validated our model externally. Thus, our results do not indicate the performance in actual clinical practice. Second, we have not adopted state-of-the-art transfer learning algorithms, such as gapBoost, distant domain transfer learning, selective learning algorithm, multilinear relationship networks, and transitive transfer learning, that have been discussed in systematic reviews [37,38]. These algorithms might yield better prediction performance. Third, we have not compared our method with other correction approaches for temporal performance drift and detection mechanisms of temporal performance drift, such as those proposed by Davis et al [1,2,39]. Fourth, we have not considered prevalent time-series models, such as recurrent neural networks and long short-term memory [40,41], as well as adding historical aggregate feature representations (eg, average laboratory test results and vital signs for the past 48 h) [42]. These methods may yield effects equivalent to those of the transfer learning approach.

### Conclusions

This study addressed the problem of performance drift in clinical prediction models. We proposed a novel transfer learning–based modeling framework and validated it using real EHR data from the University of Kansas Medical Center for AKI prediction. The proposed TransferGBM model overcomes the problems of insufficient target data and drifting data distribution through transferring old knowledge and integrating old and new knowledge models. The results showed that TransferGBM is superior to both transported and refitted models.

## Appendix

Multimedia Appendix 1 Detailed performance comparison under different experimental settings.

## Abbreviations

AdaptedGBM: adapted gradient boosting machine
AKI: acute kidney injury
AUROC: area under the receiver operating characteristic curve
DT: decision tree
EHR: electronic health record
GBM: gradient boosting machine
KNN: K-nearest neighbor
LR: logistic regression
RefittedGBM: refitted gradient boosting machine
RF: random forest
SCr: serum creatinine
SourceGBM: source gradient boosting machine
TransferGBM: transfer learning gradient boosting machine
TransportedGBM: transported gradient boosting machine

## References

[1] Davis SE, Greevy RA, Fonnesbeck C, Lasko TA, Walsh CG, Matheny ME (2019). A nonparametric updating method to correct clinical prediction model drift. J Am Med Inform Assoc.

[2] Davis SE, Lasko TA, Chen G, Siew ED, Matheny ME (2017). Calibration drift in regression and machine learning models for acute kidney injury. J Am Med Inform Assoc.

[3] Debray TPA, Vergouwe Y, Koffijberg H, Nieboer D, Steyerberg EW, Moons KGM (2015). A new framework to enhance the interpretation of external validation studies of clinical prediction models. J Clin Epidemiol.

[4] Adibi A, Sadatsafavi M, Ioannidis JPA (2020). Validation and utility testing of clinical prediction models: Time to change the approach. JAMA.

[5] Moons KGM, Kengne AP, Grobbee DE, Royston P, Vergouwe Y, Altman DG, Woodward M (2012). Risk prediction models: II. External validation, model updating, and impact assessment. Heart.

[6] Moons KGM, Altman DG, Vergouwe Y, Royston P (2009). Prognosis and prognostic research: application and impact of prognostic models in clinical practice. BMJ.

[7] Siregar S, Nieboer D, Vergouwe Y, Versteegh MI, Noyez L, Vonk AB, Steyerberg EW, Takkenberg JJ (2016). Improved prediction by dynamic modeling. Circ: Cardiovascular Quality and Outcomes.

[8] Zeng X, McMahon GM, Brunelli SM, Bates DW, Waikar SS (2014). Incidence, outcomes, and comparisons across definitions of AKI in hospitalized individuals. Clin J Am Soc Nephrol.

[9] Hoste EAJ, Bagshaw SM, Bellomo R, Cely CM, Colman R, Cruz DN, Edipidis K, Forni LG, Gomersall CD, Govil D, Honoré PM, Joannes-Boyau O, Joannidis M, Korhonen A, Lavrentieva A, Mehta RL, Palevsky P, Roessler E, Ronco C, Uchino S, Vazquez JA, Vidal Andrade E, Webb S, Kellum JA (2015). Epidemiology of acute kidney injury in critically ill patients: the multinational AKI-EPI study. Intensive Care Med.

[10] Haines RW, Lin S, Hewson R, Kirwan CJ, Torrance HD, O'Dwyer MJ, West A, Brohi K, Pearse RM, Zolfaghari P, Prowle JR (2018). Acute kidney injury in trauma patients admitted to critical care: Development and validation of a diagnostic prediction model. Sci Rep.

[11] Dai W, Chen Y, Xue G, Yang Q, Yu Y (2008). Translated learning: transfer learning across different feature spaces. NIPS'08: Proceedings of the 21st International Conference on Neural Information Processing Systems.

[12] Dai W, Yang Q, Xue G, Yu Y (2007). Boosting for transfer learning. ICML '07: Proceedings of the 24th International Conference on Machine Learning.

[13] Long M, Wang J, Ding G, Sun J, Yu P (2013). Transfer Feature Learning with Joint Distribution Adaptation.

[14] Pan SJ, Yang Q (2010). A survey on transfer learning. IEEE Trans. Knowl. Data Eng.

[15] Segev N, Harel M, Mannor S, Crammer K, El-Yaniv R (2017). Learn on source, refine on target: A model transfer learning framework with random forests. IEEE Trans. Pattern Anal. Mach. Intell.

[16] Weiss K, Khoshgoftaar TM, Wang D (2016). A survey of transfer learning. J Big Data.

[17] Wiens J, Guttag J, Horvitz E (2014). A study in transfer learning: leveraging data from multiple hospitals to enhance hospital-specific predictions. J Am Med Inform Assoc.

[18] Khwaja A (2012). KDIGO clinical practice guidelines for acute kidney injury. Nephron Clin Pract.

[19] Hsu C, Liu C, Tain Y, Kuo C, Lin Y (2020). Machine learning model for risk prediction of community-acquired acute kidney injury hospitalization from electronic health records: Development and validation study. J Med Internet Res.

[20] Song X, Yu ASL, Kellum JA, Waitman LR, Matheny ME, Simpson SQ, Hu Y, Liu M (2020). Cross-site transportability of an explainable artificial intelligence model for acute kidney injury prediction. Nat Commun.

[21] Rosenbloom ST, Carroll RJ, Warner JL, Matheny ME, Denny JC (2017). Representing knowledge consistently across health systems. Yearb Med Inform.

[22] Koyner JL, Carey KA, Edelson DP, Churpek MM (2018). The development of a machine learning inpatient acute kidney injury prediction model. Crit Care Med.

[23] Singer JD, Willett JB (2016). It’s about time: Using discrete-time survival analysis to study duration and the timing of events. Journal of Educational Statistics.

[24] He J, Hu Y, Zhang X, Wu L, Waitman LR, Liu M (2019). Multi-perspective predictive modeling for acute kidney injury in general hospital populations using electronic medical records. JAMIA Open.

[25] Kim K, Yang H, Yi J, Son H, Ryu J, Kim YC, Jeong JC, Chin HJ, Na KY, Chae D, Han SS, Kim S (2021). Real-time clinical decision support based on recurrent neural networks for in-hospital acute kidney injury: External validation and model interpretation. J Med Internet Res.

[26] Sung M, Hahn S, Han CH, Lee JM, Lee J, Yoo J, Heo J, Kim YS, Chung KS (2021). Event prediction model considering time and input error using electronic medical records in the intensive care unit: Retrospective study. JMIR Med Inform.

[27] Wei C, Zhang L, Feng Y, Ma A, Kang Y (2022). Machine learning model for predicting acute kidney injury progression in critically ill patients. BMC Med Inform Decis Mak.

[28] Wolpert DH (1992). Stacked generalization. Neural Networks.

[29] Jiménez-Valverde A (2012). Insights into the area under the receiver operating characteristic curve (AUC) as a discrimination measure in species distribution modelling. Global Ecology and Biogeography.

[30] Guidance Regarding Methods for De-identification of Protected Health Information in Accordance with the Health Insurance Portability and Accountability Act (HIPAA) Privacy Rule. US Department of Health and Human Services, Office for Human Research Protections.

[31] Pedregosa F, Varoquaux G, Gramfort A, Michel V, Thirion B, Grisel O, Blondel M (2011). Scikit-learn: Machine Learning in Python. The Journal of Machine Learning Research.

[32] Corradi JP, Thompson S, Mather JF, Waszynski CM, Dicks RS (2018). Prediction of incident delirium using a random forest classifier. J Med Syst.

[33] Kulkarni VY, Sinha PK, Petare MC (2015). Weighted hybrid decision tree model for random forest classifier. J. Inst. Eng. India Ser. B.

[34] Chen T, He T, Benesty M, Khotilovich V, Tang Y, Cho H (2015). XGBoost: eXtreme Gradient Boosting, R package version 04-2. R Project.

[35] Chen T, Guestrin C (2016). XGBoost: A Scalable Tree Boosting System. KDD '16: Proceedings of the 22nd ACM SIGKDD International Conference on Knowledge Discovery and Data Mining.

[36] Ogunleye A, Wang Q (2020). XGBoost model for chronic kidney disease diagnosis. IEEE/ACM Trans Comput Biol Bioinform.

[37] Zhuang F, Qi Z, Duan K, Xi D, Zhu Y, Zhu H, Xiong H, He Q (2021). A comprehensive survey on transfer learning. Proc. IEEE.

[38] Niu S, Liu Y, Wang J, Song H (2020). A decade survey of transfer learning (2010–2020). IEEE Trans. Artif. Intell.

[39] Davis SE, Greevy RA, Lasko TA, Walsh CG, Matheny ME (2020). Detection of calibration drift in clinical prediction models to inform model updating. J Biomed Inform.

[40] Shickel B, Tighe PJ, Bihorac A, Rashidi P (2018). Deep EHR: A survey of recent advances in deep learning techniques for electronic health record (EHR) analysis. IEEE J Biomed Health Inform.

[41] Yadav P, Steinbach M, Kumar V, Simon G (2018). Mining Electronic Health Records (EHRs). ACM Comput. Surv.

[42] Tomašev N, Glorot X, Rae JW, Zielinski M, Askham H, Saraiva A, Mottram A, Meyer C, Ravuri S, Protsyuk I, Connell A, Hughes CO, Karthikesalingam A, Cornebise J, Montgomery H, Rees G, Laing C, Baker CR, Peterson K, Reeves R, Hassabis D, King D, Suleyman M, Back T, Nielson C, Ledsam JR, Mohamed S (2019). A clinically applicable approach to continuous prediction of future acute kidney injury. Nature.

