# Supplementary material for: A Transfer Learning Approach to Correct the Temporal Performance Drift of Clinical Prediction Models: Retrospective Cohort Study
Source: JMIR Med Inform. 2022 Nov 9;10(11):e38053. doi: 10.2196/38053 (PMC9685506; doi:10.2196/38053)
Supplement: Multimedia Appendix 1 [file medinform_v10i11e38053_app1.docx]

**Multimedia Appendix 1.** Detailed performance comparison under different experimental settings.

| Year and model | | Any AKI^a^ stage, AUROC^b^ (95% CI) for different training set sizes | | | | | AKI stage ≥2, AUROC (95% CI) for different training set sizes | | | | | AKI stage 3, AUROC (95% CI) for different training set sizes | | | |
| --- | --- | --- | --- | --- | --- | --- | --- | --- | --- | --- | --- | --- | --- | --- | --- |
|  |  | 25% | 50% | 75% | 100% | 25% | | 50% | 75% | 100% | 25% | | 50% | 75% | 100% |
| **2012** | |  |  |  |  |  | |  |  |  |  | |  |  |  |
|  | TransportedGBM^c^ | 0.759 (0.751-0.786) | 0.759 (0.751-0.786) | 0.759 (0.751-0.786) | 0.759 (0.751-0.786) | 0.822 (0.806-0.858) | | 0.822 (0.806-0.859) | 0.822 (0.806-0.860) | 0.822 (0.806-0.861) | 0.933 (0.926-0.963) | | 0.933 (0.926-0.964) | 0.933 (0.926-0.965) | 0.933 (0.926-0.966) |
|  | RefittedGBM^d^ | 0.721 (0.713-0.750) | 0.748 (0.742-0.774) | 0.769 (0.761-0.796) | 0.759 (0.755-0.786) | 0.814 (0.799-0.855) | | 0.828 (0.814-0.867) | 0.833 (0.821-0.871) | 0.857 (0.846-0.894) | 0.894 (0.883-0.948) | | 0.904 (0.894-0.951) | 0.947 (0.943-0.976) | 0.926 (0.918-0.958) |
|  | TransferGBM^e^ | 0.759 (0.732-0.766) | 0.774 (0.746-0.782) | 0.790 (0.764-0.795) | 0.783 (0.757-0.792) | 0.830 (0.795-0.851) | | 0.844 (0.808-0.862) | 0.849 (0.818-0.870) | 0.866 (0.835-0.877) | 0.938 (0.910-0.960) | | 0.940 (0.912-0.964) | 0.970 (0.946-0.979) | 0.959 (0.932-0.973) |
| **2013** | |  |  |  |  |  | |  |  |  |  | |  |  |  |
|  | TransportedGBM | 0.784 (0.775-0.810) | 0.784 (0.775-0.810) | 0.784 (0.775-0.810) | 0.784 (0.775-0.810) | 0.811 (0.794-0.855) | | 0.811 (0.794-0.856) | 0.811 (0.794-0.857) | 0.811 (0.794-0.858) | 0.912 (0.904-0.956) | | 0.912 (0.904-0.957) | 0.912 (0.904-0.958) | 0.912 (0.904-0.959) |
|  | RefittedGBM | 0.766 (0.758-0.795) | 0.780 (0.773-0.807) | 0.792 (0.786-0819) | 0.801 (0.794-0.829) | 0.856 (0.845-0.889) | | 0.837 (0.824-0.875) | 0.868 (0.859-0.904) | 0.862 (0.852-0.897) | 0.890 (0.879-0.938) | | 0.861 (0.847-0.923) | 0.913 (0.905-0.947) | 0.900 (0.891-0.936) |
|  | TransferGBM | 0.801 (0.775-0.808) | 0.807 (0.781-0.816) | 0.816 (0.789-0.824) | 0.822 (0.794-0.830) | 0.870 (0.839-0.889) | | 0.851 (0.816-0.873) | 0.889 (0.859-0.904) | 0.883 (0.851-0.901) | 0.920 (0.890-0.936) | | 0.911 (0.880-0.935) | 0.940 (0.912-0.954) | 0.942 (0.915-0.954) |
| **2014** | |  |  |  |  |  | |  |  |  |  | |  |  |  |
|  | TransportedGBM | 0.789 (0.784-0.814) | 0.789 (0.784-0.815) | 0.789 (0.784-0.816) | 0.789 (0.784-0.817) | 0.881 (0.872-0916) | | 0.881 (0.872-0917) | 0.881 (0.872-0918) | 0.881 (0.872-0919) | 0.939 (0.933-0.973) | | 0.939 (0.933-0.974) | 0.939 (0.933-0.975) | 0.939 (0.933-0.976) |
|  | RefittedGBM | 0.756 (0.750-0.783) | 0.786 (0.780-0.813) | 0.791 (0.786-0.820) | 0.803 (0.797-0.829) | 0.896 (0.888-0.929) | | 0.907 (0.899-0.937) | 0.890 (0.881-0.924) | 0.895 (0.886-0.929) | 0.913 (0.906-0.953) | | 0.904 (0.895-0.947) | 0.907 (0.899-0.944) | 0.933 (0.926-0.971) |
|  | TransferGBM | 0.793 (0.766-0.800) | 0.815 (0.788-0.822) | 0.818 (0.792-0.824) | 0.828 (0.802-0.834) | 0.921 (0.893-0.932) | | 0.931 (0.905-0.941) | 0.914 (0.886-0.928) | 0.917 (0.888-0.929) | 0.943 (0.917-0.968) | | 0.935 (0.907-0.953) | 0.935 (0.907-0.952) | 0.954 (0.927-0.977) |
| **2015** | |  |  |  |  |  | |  |  |  |  | |  |  |  |
|  | TransportedGBM | 0.759 (0.751-0.786) | 0.759 (0.751-0.787) | 0.759 (0.751-0.788) | 0.759 (0.751-0.789) | 0.868 (0.859-0901) | | 0.868 (0.859-0902) | 0.868 (0.859-0903) | 0.868 (0.859-0904) | 0.907 (0.899-0.940) | | 0.907 (0.899-0.950) | 0.907 (0.899-0.960) | 0.907 (0.899-0.970) |
|  | RefittedGBM | 0.774 (0.767-0.800) | 0.790 (0.784-0.817) | 0.794 (0.789-0.820) | 0.794 (0.787-0.820) | 0.899 (0.892-0.933) | | 0.902 (0.895-0.933) | 0.919 (0.914-0.954) | 0.918 (0.912-0.947) | 0.927 (0.920-0.960) | | 0.921 (0.915-0.956) | 0.922 (0.916-0.956) | 0.927 (0.920-0.961) |
|  | TransferGBM | 0.804 (0.778-0.812) | 0.813 (0.787-0.820) | 0.818 (0.792-0.824) | 0.818 (0.791-0.824) | 0.912 (0.884-0.925) | | 0.916 (0.888-0.930) | 0.943 (0.917-0.951) | 0.946 (0.920-0.959) | 0.941 (0.915-0.956) | | 0.941 (0.914-0.955) | 0.941 (0.914-0.959) | 0.948 (0.922-0.965) |
| **2016** | |  |  |  |  |  | |  |  |  |  | |  |  |  |
|  | TransportedGBM | 0.745 (0.738-0.771) | 0.745 (0.738-0.772) | 0.745 (0.738-0.773) | 0.745 (0.738-0.774) | 0.827 (0.814-0.860) | | 0.827 (0.814-0.870) | 0.827 (0.814-0.880) | 0.827 (0.814-0.890) | 0.886 (0.877-0.923) | | 0.886 (0.877-0.924) | 0.886 (0.877-0.925) | 0.886 (0.877-0.926) |
|  | RefittedGBM | 0.762 (0.758-0.789) | 0.772 (0.766-0.800) | 0.775 (0.770-0.801) | 0.777 (0.771-0.802) | 0.842 (0.831-0.879) | | 0.860 (0.849-0.892) | 0.863 (0.852-0.896) | 0.878 (0.869-0.910) | 0.880 (0.869-0.929) | | 0.869 (0.857-0.911) | 0.859 (0.846-0.908) | 0.862 (0.850-0.910) |
|  | TransferGBM | 0.784 (0.758-0.791) | 0.795 (0.770-0.801) | 0.799 (0.773-0.805) | 0.800 (0.775-0.807) | 0.856 (0.823-0.874) | | 0.873 (0.842-0.887) | 0.879 (0.850-0.891) | 0.892 (0.862-0.905) | 0.920 (0.892-0.940) | | 0.893 (0.862-0.918) | 0.892 (0.861-0.923) | 0.886 (0.854-0.911) |
| **2017** | |  |  |  |  |  | |  |  |  |  | |  |  |  |
|  | TransportedGBM | 0.751 (0.745-0.779) | 0.751 (0.745-0.780) | 0.751 (0.745-0.781) | 0.751 (0.745-0.782) | 0.848 (0.834-0.886) | | 0.848 (0.834-0.887) | 0.848 (0.834-0.888) | 0.848 (0.834-0.889) | 0.937 (0.930-0.969) | | 0.937 (0.930-0.970) | 0.937 (0.930-0.971) | 0.937 (0.930-0.972) |
|  | RefittedGBM | 0.753 (0.745-0.780) | 0.768 (0.761-0.795) | 0.777 (0.770-0.804) | 0.779 (0.772-0.806) | 0.853 (0.841-0.888) | | 0.868 (0.857-0.906) | 0.878 (0.869-0.912) | 0.899 (0.891-0.930) | 0.922 (0.915-0.965) | | 0.939 (0.934-0.978) | 0.937 (0.931-0.974) | 0.926 (0.918-0.958) |
|  | TransferGBM | 0.781 (0.754-0.788) | 0.792 (0.765-0.800) | 0.800 (0.773-0.806) | 0.800 (0.774-0.809) | 0.880 (0.848-0.895) | | 0.902 (0.873-0.913) | 0.907 (0.878-0.922) | 0.921 (0.893-0.934) | 0.948 (0.921-0.962) | | 0.955 (0.920-0.971) | 0.958 (0.932-0.968) | 0.952 (0.926-0.964) |

^a^AKI: acute kidney injury.

^b^AUROC: area under the receiver operating characteristic curve.

^c^TransportedGBM: transported gradient boosting machine.

^d^RefittedGBM: refitted gradient boosting machine.

^e^TransferGBM: transfer learning gradient boosting machine (proposed predictive modeling framework).
